# Shortage of Obesity Medicine Specialists in the United States

**DOI:** 10.1016/j.mayocp.2025.10.015

**Published:** 2026-01-06

**Authors:** Simar Singh Bajaj, Shreyas Teegala, Fatima Cody Stanford

**Affiliations:** a Stanford University School of Medicine, Palo Alto, CA; b Vanderbilt University, Nashville, TN; c Harvard Medical School, Boston, MA

Obesity is among the most prevalent, expensive diseases in the United States, affecting more than 100 million Americans and costing $1.72 trillion annually.^1^ In June 2021, the Food and Drug Administration approved semaglutide for obesity, bringing increased attention to the obesity medicine field. In recent years, the growth of the specialty has accelerated, with physicians pursuing fellowship training or 60 continuing medical education credits, followed by an examination, for board certification.

Previous research suggests that patients seen by obesity medicine specialists are more likely to receive evidence-based weight management treatments and to achieve 10% or more weight loss than those treated by general primary care physicians.^2^

## Objective

Given limited research on board-certified obesity medicine physicians, we sought to characterize their geographic distribution across US counties and to evaluate the association between obesity prevalence and their absence while adjusting for sociodemographic factors.

## Methods

On December 30, 2024, a list of 9895 board-certified obesity medicine physicians and their practice locations was collected from the American Board of Obesity Medicine. The subset that was fellowship trained was identified from the Obesity Medicine Fellowship Council directory, which lists graduates of accredited clinical obesity medicine fellowships across the country. Physicians practicing outside the 50 states and District of Columbia were excluded. Counties were stratified by the absence of a board-certified obesity medicine physician and linked to the 2024 County Health Rankings & Roadmaps data set.

Potential confounders included race, ethnicity, sex, education, health professional shortage area, median household income (low, medium, high per tertiles), rurality, insurance, and unemployment. They were screened by linear regression for associations with county-level obesity percentage (exposure) and the Mann-Whitney test for associations with the absence of a board-certified obesity medicine physician (outcome). We then conducted stepwise logistic regression, with data presented as average marginal effects with 95% CIs.^3^ Because all data were publicly available, informed consent was not required, and the institutional review board exempted this study.

## Results

In 2024, there were 9423 board-certified obesity medicine physicians in the United States, with half (n=4718 [50.1%]) certified from 2022 to 2024. Most specialists came from primary care backgrounds (n=6876 [73.0%]). Only 129 were fellowship trained, with most (n=68 [52.7%]) practicing in 5 states—New York, Pennsylvania, Massachusetts, California, and Ohio. In total, 44% of states had no fellowship-trained obesity specialist.

In the full cohort, board-certified obesity medicine physicians practiced across 1003 of 3143 (31.9%) counties and all 50 states (Figure). County-level percentages of women and unemployment were not associated with the exposure and outcome, respectively, so neither was evaluated as a confounder. Univariable analyses indicated that counties without a board-certified obesity medicine physician had higher obesity prevalence (38.3% vs 35.8%), rural residents (99.4% vs 29.5%), and median household incomes in the lowest tertile (41.6% vs 15.7%; Table).

In multivariable logistic regression adjusting for American Indian/Alaska Natives, Asian Americans, Black Americans, high school graduates, health professional shortage area, median household income, rurality, and lack of insurance, a 1 percentage point increase in county-level obesity was associated with 0.70 percentage point increase (95% CI, 0.33 to 1.08) in the probability that a county had no board-certified obesity medicine physician.

## Discussion

We highlight significant geographic disparities in access to specialty obesity care, with more than two-thirds of US counties not having a board-certified obesity medicine physician. Whereas these counties were predominantly rural and less wealthy, increased prevalence of obesity was one of the strongest independent predictors for the absence of a specialist, suggesting that the highest need areas were the least served.

As with many specialties, physician compensation and insurance reimbursement may help explain these geographic inequities. Primary care physicians can certainly manage patients with obesity, but limited training, short patient visits, and competing priorities may hinder their ability to offer comprehensive care.^2^ On average, medical schools dedicate 10 hours to obesity education,^4^ and only 2.5% of internal medicine residency leaders believed their trainees were “very prepared” to manage obesity.^5^ As obesity education continues to be strengthened for students and trainees, promoting greater utilization of the continuing medical education pathway, which does not require leave from practice, may empower attendings to provide more evidence-based obesity care.^2,6^ Leveraging telemedicine may also expand access to care in counties without obesity specialists.

This study has limitations. First, we used the publicly available American Board of Obesity Medicine directory to determine practice locations, so physicians who recently moved or work across multiple counties may have been imprecisely classified. Second, the 2024 county data set imputed obesity percentages with 2021 values and therefore may not reflect recent changes in prevalence.

## Conclusion

Board-certified obesity medicine physicians are disproportionately absent in counties with the highest obesity rates, revealing a critical mismatch between burden and access to care. These findings underscore the need for workforce interventions and targeted policies to improve availability of obesity care to those who need it.

## Figures and Tables

**FIGURE.**
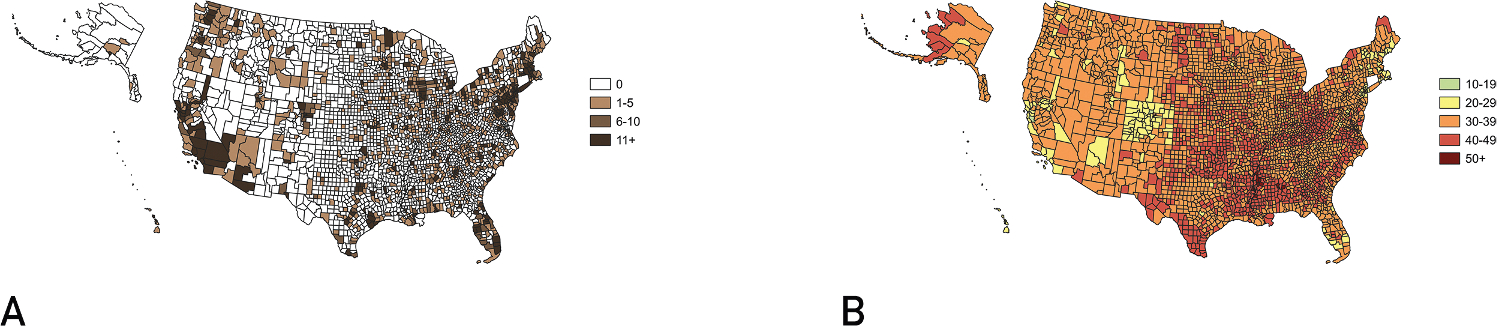
A, Number of board-certified obesity medicine physicians by US county, 12024. B, Obesity rate by US county, 2024 (%).

**TABLE.** Baseline Characteristics of Counties With and Without a Board-Certified Obesity Medicine Physician and Stepwise Logistic Regression Model to Predict the Absence of a Board-Certified Obesity Medicine Physician^a^

	Univariable analysis			Multivariable analysis	
	Board-certified obesity medicine physician in a county (n= 1003)	No board-certified obesity medicine physician in a county (n=2140)		Unique predictors for the absence of board-certified obesity medicine physician in a county (N=3143)	
	Median (interquartile range) or count (%)		*P* value	Odds ratio (95% CI)	*P* value

Obesity, %	35.8 (32.2–38.8)	38.3 (36.2–40.8)	<.001	1.06 (1.03–1.09)	<.001
Race, ethnicity, sex					
AIAN, %	0.6 (0.4–1.1)	0.8 (0.5–1.7)	<.001	1.00 (0.98–1.02)	.83
Asian, %	1.7 (0.9–3.6)	0.7 (0.5–1.0)	<.001	0.95 (0.90–0.99)	.018
Black, %	4.9 (1.7–13.1)	1.6 (0.7–7.8)	<.001	0.99 (0.98–1.00)	.026
Hispanic, %	6.9 (3.8–14.1)	4.3 (2.6–9.1)	<.001	N/A^b^	N/A^b^
White, %	76.8 (60.0–87.4)	84.7 (64.2–92.4)	<.001	N/A^b^	N/A^b^
Women, %	50.3 (49.7–51.0)	49.6 (48.7–50.4)	<.001	N/A^c^	N/A^c^
Social position					
High school graduate, %	91.1 (88.3–93.3)	88.4 (84.0–92.0)	<.001	0.98 (0.96–1.01)	.18
HPSA	759 (75.7)	1882 (87.9)	<.001	1.02 (0.78–1.34)	.86
Median household income					
Low	157 (15.7)	891 (41.6)		Reference	Reference
Medium	299 (29.8)	749 (35.0)	<.001	0.75 (0.57–0.99)	.045
High	547 (54.5)	500 (23.4)		0.56 (0.41–0.76)	<.001
Rural, %	29.5 (13.1–52.6)	99.4 (57.5–100.0)	<.001	1.04 (1.04–1.05)	<.001
Unemployed, %	3.4 (2.8–4.1)	3.4 (2.7–4.3)	.48	N/A^d^	N/A^d^
Uninsured, %	8.6 (6.5–12.6)	11.2 (8.1–15.3)	<.001	1.04 (1.01–1.07)	.003

a AIAN, American Indian/Alaska Native; HPSA, health professional shortage area; N/A, not applicable.

b Variable was excluded because it was not significantly associated with the outcome in the multivariable model.

c Variable was excluded because it was not significantly associated with the exposure in univariable linear regression (not shown).

d Variable was excluded because it was not significantly associated with the outcome in the univariable Mann-Whitney test.
